# Influence of Mono- and Bimetallic PtO_x_, PdO_x,_ PtPdO_x_ Clusters on CO Sensing by SnO_2_ Based Gas Sensors

**DOI:** 10.3390/nano8110917

**Published:** 2018-11-07

**Authors:** Pavel Kutukov, Marina Rumyantseva, Valeriy Krivetskiy, Darya Filatova, Maria Batuk, Joke Hadermann, Nikolay Khmelevsky, Anatoly Aksenenko, Alexander Gaskov

**Affiliations:** 1 Chemistry Department, Moscow State University, 119991 Moscow, Russia; spamflame@gmail.com (P.K.); vkrivetsky@gmail.com (V.K.); gak1.analyt@gmail.com (D.F.); 2 EMAT, University of Antwerp, B-2020 Antwerp, Belgium; maria.batuk@uantwerpen.be (M.B.); Joke.Hadermann@uantwerpen.be (J.H.); 3 LISM, Moscow State Technological University Stankin, 127055 Moscow, Russia; khmelevsky@mail.ru (N.K.); a.aksenenko@lism-stankin.ru (A.A.)

**Keywords:** nanocrystalline semiconductor oxides, nanocomposites, tin oxide, platinum, palladium, bimetallic particles, carbon monoxide, gas sensor, response inversion

## Abstract

To obtain a nanocrystalline SnO_2_ matrix and mono- and bimetallic nanocomposites SnO_2_/Pd, SnO_2_/Pt, and SnO_2_/PtPd, a flame spray pyrolysis with subsequent impregnation was used. The materials were characterized using X-ray diffraction (XRD), a single-point BET method, transmission electron microscopy (TEM), and high angle annular dark field scanning transmission electron microscopy (HAADF-STEM) with energy dispersive X-ray (EDX) mapping. The electronic state of the metals in mono- and bimetallic clusters was determined using X-ray photoelectron spectroscopy (XPS). The active surface sites were investigated using the Fourier Transform infrared spectroscopy (FTIR) and thermo-programmed reduction with hydrogen (TPR-H_2_) methods. The sensor response of blank SnO_2_ and nanocomposites had a carbon monoxide (CO) level of 6.7 ppm and was determined in the temperature range 60–300 °C in dry (Relative Humidity (RH) = 0%) and humid (RH = 20%) air. The sensor properties of the mono- and bimetallic nanocomposites were analyzed on the basis of information on the electronic state, the distribution of modifiers in SnO_2_ matrix, and active surface centers. For SnO_2_/PtPd, the combined effect of the modifiers on the electrophysical properties of SnO_2_ explained the inversion of sensor response from *n*- to *p*-types observed in dry conditions.

## 1. Introduction

Because SnO_2_ is a wide-bandgap oxygen-deficient *n*-type semiconductor with optical transparency, electron conductivity, and a high specific surface area, it is suitable for a large range of applications, including in solar cells, as catalytic support, and as solid state gas sensors [1]. However, the use of bare SnO_2_ is often limited by lack of selectivity and a high operating temperature [2]. Chemical modification is a well-established practice intended to solve those problems [2,3,4,5]. This involves the creation of new active sites, with specific adsorptivity and reactivity toward target gases (i.e., carbon monoxide), on the surface of a semiconductor matrix.

Carbon monoxide (CO) is a colorless, odorless, and tasteless toxic gas, produced by automotive emissions, natural gas manufacturing, industrial activities, and the incomplete burning of fuels [6]. In the human body, it reacts readily with hemoglobin to form carboxyhemoglobin. Carbon monoxide exposure is still one of the leading causes of unintentional and suicidal poisonings, and it causes a large number of deaths annually. [7] Thus, real-time monitoring of CO is extremely important for safety reasons.

Carbon monoxide is a reducing gas without pronounced acid or basic properties [3]. To enhance the sensor signal of a SnO_2_ based sensor toward such gases, the catalytically active additives metallic platinum, gold, and silver are the most effective [6,8]. These modifiers take part in the oxidation of CO on the surface of the semiconductor oxide and lead to a change in the type and concentration of active groups on that surface [5]. This not only leads to an increased sensor response, but also to a decrease in temperature, at which a maximum sensitivity is observed [2,3,4,5]. The selectivity of heterogeneous catalysts of oxidative processes is determined by the energy of adsorption of the reducing gas, the binding energy with surface oxygen (which is an oxidizer) and binding energies with intermediates and reaction products. In CO oxidation, the optimal catalysts are palladium (Pd) and platinum (Pt), since the energy of chemisorption of oxygen on the clusters of these metals (340–360 kJ/mol) is close to the binding energy of CO with their surface [9,10]. Palladium and platinum are some of the most effective modifiers for improving the sensor properties of semiconductor metal oxides in CO detection [11,12,13,14,15,16,17,18,19,20,21,22,23,24,25,26,27].

The properties of bimetallic catalysts are now being actively investigated due to the expected synergistic effect—i.e., that the activity of the bimetallic catalyst exceeds the sum of the activities of its monometallic analogues [28,29,30,31,32,33,34,35,36,37,38,39,40]. In gas sensors, it is thought that the surface modification of semiconductor oxides with bimetallic catalysts should create optimal conditions for both electron and ion exchange (spillover effect) between the surface nanoclusters and the metal oxide support [41]. As bimetallic catalytically active modifiers for gas sensors, nanoparticles that contain platinum group metals in various combinations are usually studied, as well as Au and *d*-elements such as Fe, Co, Ni, and Cu [42,43,44,45,46,47,48,49,50,51,52]. Despite the fact that numerous monometallic systems have already been studied, including SnO_2_/Pt and SnO_2_/Pd, only a few reports on CO sensing properties of bimetallic SnO_2_/PtPd nanocomposite are known [44,52].

The procedure of conventional and widespread wet-chemical synthesis of nanocrystalline SnO_2_ is well-known and relatively simple [53]. However, this method is rather time-consuming, as the hydrated SnO_2_ needs to be repeatedly washed. Flame spray pyrolysis (FSP) is an alternative method for producing metal oxides for catalysts and semiconductor gas sensors [54,55,56]. The advantage of the FSP method is the ability to produce highly dispersed materials with nanometer-sized crystallites that have high crystallinity and porosity. The use of precursors of the main components and modifiers during the one-stage synthesis allows for the obtainment of a modified material with the necessary set of functional properties regarding the specific surface area, electrical conductivity, acid-base, and oxidation-reduction ability in interaction with gases [17,57,58,59,60,61,62,63,64].

In this study, the nanocrystalline SnO_2_ matrix was synthesized using FSP. Mono- and bimetallic nanocomposites SnO_2_/Pd, SnO_2_/Pt, and SnO_2_/PtPd were then obtained via impregnation. The sample characteristics are presented in Table 1. The gas sensing properties of the bimetallic nanocomposite SnO_2_/PtPd were compared to those of the monometallic nanocomposites SnO_2_/Pt and SnO_2_/Pd and analyzed on the basis of information on the Pt and Pd electronic state, the distribution of modifiers in the SnO_2_ matrix, and the type and concentration of active centers on the surface of the nanocomposites.

## 2. Results and Discussion

**Figure 1:**
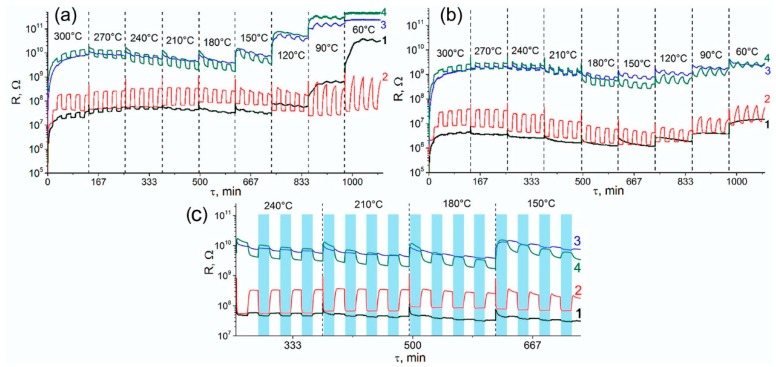
Resistance of the samples in the temperature range 60–300 °C under the periodic change of the gas phase composition at relative humidity (**a**) Relative Humidity (RH) = 0% and (**b**) RH = 20%. (**c**) An enlarged image of the resistance variation of the samples when measured in dry air (RH = 0%). (1) SnO_2_, (2) SnO_2_/Pd, (3) SnO_2_/Pt, (4) SnO_2_/PtPd. Pale blue areas correspond to exposure in CO.

**Figure 2:**
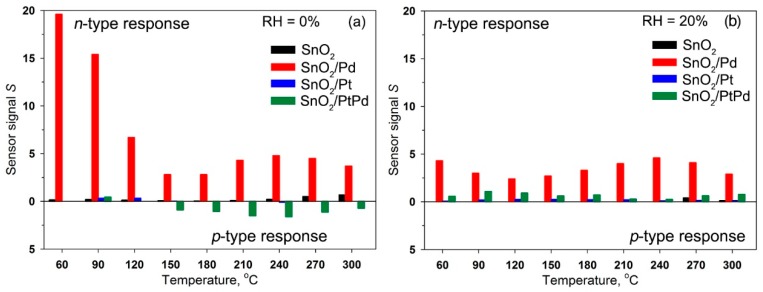
Sensor signal to 6.7 ppm CO of blank SnO_2_ and mono- and bimetallic nanocomposites in the temperature range 60–300 °C at relative humidity RH = 0% (**a**) and RH = 20% (**b**).

Figure 1 shows the change in the resistance of the samples in the temperature range 60–300 °C under periodic change of the gas phase composition: air (15 min), 6.7 ppm CO in air (15 min). The measurements were effectuated in dry air (Relative Humidity (RH) = 0%, Figure 1a) and humid air (RH = 20%, Figure 1b). The decrease in the electrical resistance in the presence of CO (*n*-type response) was due to the oxidation of carbon monoxide by chemisorbed oxygen:

(1) $$\beta \cdot {\mathrm{CO}}_{\left(gas\right)}+O_{\beta \left(ads\right)}^{-\alpha }↔\beta \cdot {\mathrm{CO}}_{2\left(gas\right)}+\alpha \cdot e^{-}$$

where ${\mathrm{CO}}_{\left(gas\right)}$ represents the carbon monoxide molecule in the gas phase, $O_{\beta \left(ads\right)}^{-\alpha }$ is chemisorbed oxygen, ${\mathrm{CO}}_{2\left(gas\right)}$ is the reaction product desorbed to the gas phase, and *e* is an electron injected into the conduction band of the *n*-type semiconductor. The sensor signal for *n*-type response was calculated for each temperature as $S=\frac{\Delta G}{G_{air}}=\frac{G_{gas}-G_{air}}{G_{air}}$, where *G_air_* is the sample’s conductance in air and *G_gas_* is the sample’s conductance in the presence of 6.7 ppm CO in air (Figure 2). For SnO_2_/PtPd nanocomposite the increase in the electrical resistance in the presence of CO (*p*-type response) was observed in the temperature range 150–300 °C at RH = 0% (Figure 1c). In these cases, the sensor signal was calculated as $S=\frac{\Delta R}{R_{air}}=\frac{R_{gas}-R_{air}}{R_{air}}$, where *R_air_* is the sample’s resistance in air and *R_gas_* is the sample’s resistance in the presence of 6.7 ppm CO in air.

The data presented in Figure 1 and Figure 2, revealed the following trends.

(i) Comparison of the resistance values show that the introduction of modifiers caused an increase in the resistance of tin dioxide. This effect was most pronounced for nanocomposites containing platinum.
(ii) For the non-modified SnO_2_, the value of the *n*-type sensor response toward CO increased with increasing measurement temperature and reached a maximum at 270–300 °C. The increase in humidity almost completely suppressed the sensor response of SnO_2_ sample.
(iii) For the SnO_2_/Pd nanocomposite, only the *n*-type response was observed. Two maxima can be distinguished on the temperature dependence of the sensor signal: one in the temperature range 240–270 °C and one in the range 60–90 °C. The sensor signal values at 240–270 °C decreased slightly with increasing air humidity from RH = 0% to RH = 20%. In the low-temperature interval, an increase in humidity led to a significant decrease in the sensor signal.
(iv) The SnO_2_/Pt nanocomposite exhibited a low sensor response. However, in the low-temperature range, its response to CO in dry air exceeded the analogous value for unmodified SnO_2_, while in the high-temperature region its sensor signal turns out to be lower than for SnO_2._ The inversion of the sensor response from the *n*-type to the *p*-type is observed only when measured in dry air at T = 240 °C.
(v) When performing the measurements in dry air, the inversion of the sensor response was characteristic for bimetallic nanocomposite SnO_2_/PtPd over a wide temperature range. The maximum of the *p*-type response was observed at 210–240 °C. The increase in air humidity led to the disappearance of the inversion of the response. The observed *n*-type response in the whole temperature range was lower than for palladium containing monometallic nanocomposite SnO_2_/Pd.

To determine the factors responsible for the formation of the sensor response of mono- and bimetallic nanocomposites, the phase composition, the electronic state of platinum and palladium, and their distribution in the SnO_2_ matrix were investigated. The effect of the presence of various modifiers on the surface composition of nanocrystalline SnO_2_ was studied in detail.

**Figure 3:**
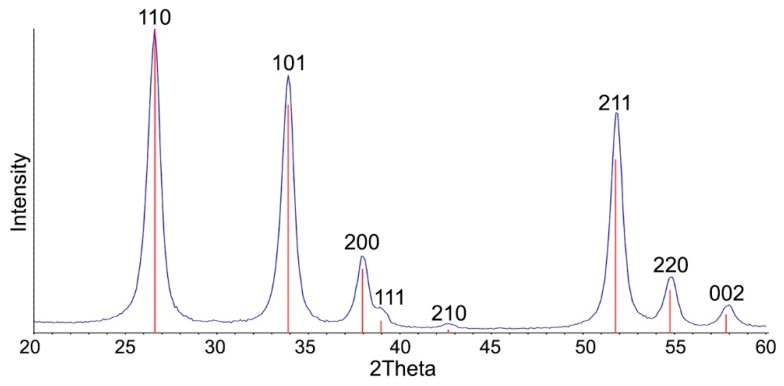
X-ray diffraction (XRD) pattern of SnO_2_ powder obtained using the flame spray pyrolysis (FSP) method. Vertical lines correspond to the ICDD 41-1445 reference (SnO_2_ cassiterite).

The X-ray diffraction (XRD) pattern of SnO_2_ corresponds to the cassiterite phase (ICDD 41-1445, Figure 3). The introduction of catalytic modifiers did not led to a change in the phase composition of the samples. The reflections corresponding to Pt- or Pd-containing phases do not appear on the diffractograms of the nanocomposites.

**Figure 4:**
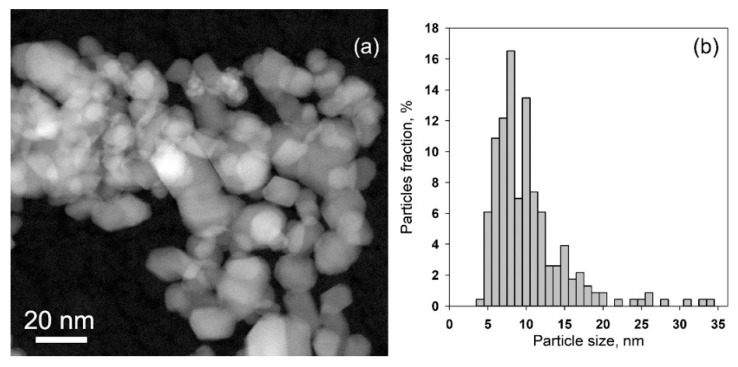
High angle annular dark field scanning transmission electron microscopy (HAADF-STEM) image: (**a**) and particle size distribution (**b**) of the SnO_2_ matrix.

**Figure 5:**
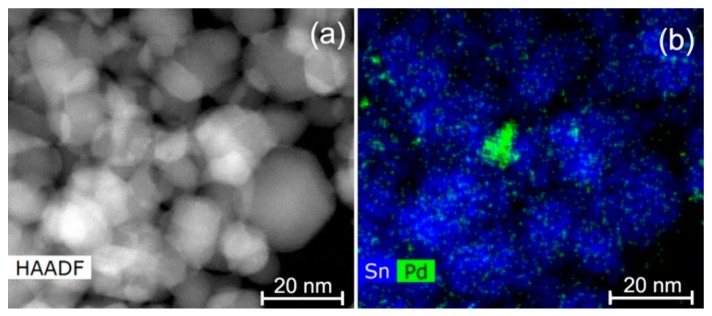
HAADF-STEM image: (**a**) energy dispersive X-ray scanning transmission electron microscopy (EDX-STEM) map (**b**) of the SnO_2_/Pd nanocomposite.

According to the high angle annular dark field scanning transmission electron microscopy (HAADF-STEM) images, all the materials were composed of agglomerated crystalline SnO_2_ nanoparticles, with sizes varying from approximately 5–35 nm, with an average size of 10.7 ± 4.9 nm (Figure 4). The introduction of modifiers does not affect the particle size of SnO_2_. In SnO_2_/Pd nanocomposite (Figure 5) several Pd particles with a size 8–20 nm were found among the SnO_2_ matrix particles (Figure 5b). Also small Pd particles with a size of 2 nm could be seen on the images and energy dispersive X-ray (EDX) maps (Figure 5b).

**Figure 6:**
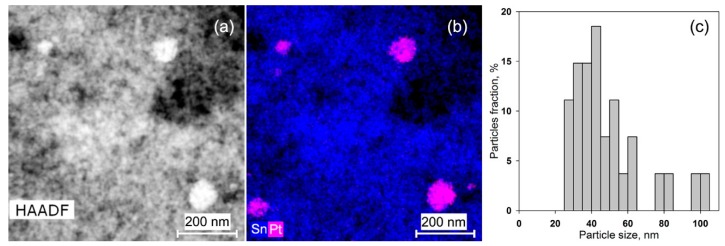
HAADF-STEM image: (**a**) EDX-STEM map (**b**) and Pt particles size distribution (**c**) of the SnO_2_/Pt nanocomposite.

In the SnO_2_/Pt nanocomposite, the Pt particles were very easy to see on the HAADF-STEM images (Figure 6a) because they are large and bright. Their size varied in the range of 25–100 nm and the average size was 49.1 ± 19.5 nm (Figure 6c).

**Figure 7:**
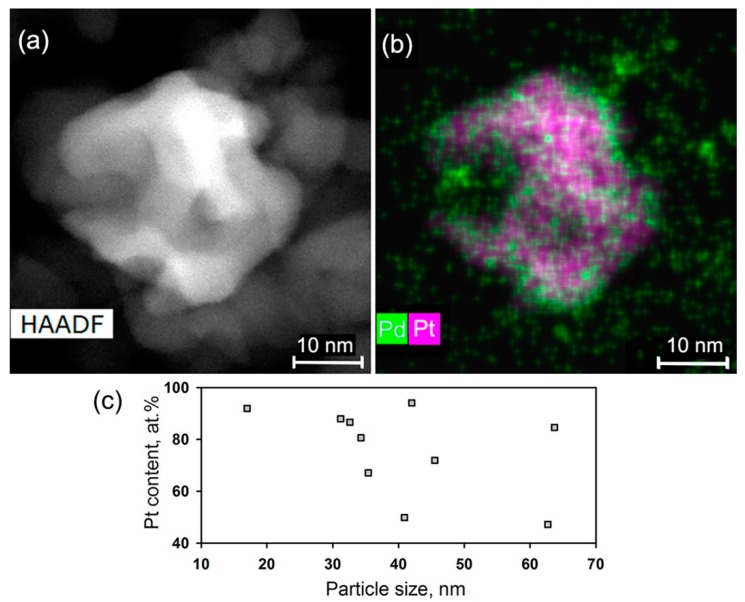
HAADF-STEM image: (**a**) EDX-STEM map (**b**) of the SnO_2_/PtPd nanocomposite. (**c**) Pt content in PtPd nanoparticles of different size.

The nanocomposite SnO_2_/PtPd contained bimetallic nanoparticles particles, which were present in a form of agglomerates with the size 17–64 nm (Figure 7). According to the EDX maps, the particles contained both metals (Figure 7b), however their ratio varied from particle to particle and was independent from the particle size (Figure 7c). The distribution of Pt and Pd inside the particle was not uniform. Besides the PtPd particles, small Pd particles with a size about 2 nm could be seen on the maps (Figure 7b).

**Figure 8:**
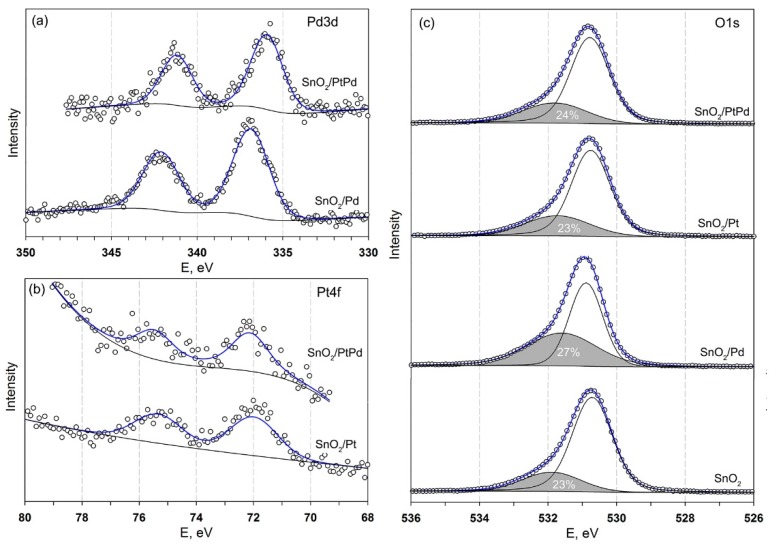
XP spectra Pd3d, (**a**) Pt4f, (**b**) O1s (**c**) of the samples.

The Pd3d and Pt4f X-ray photoelectron spectroscopy (XPS) signals of the nanocomposites SnO_2_/Pd, SnO_2_/Pt, and SnO_2_/PtPd could be fitted by only one doublet component (Figure 8a,b). Table 2 presents the Pd 3d_5/2_ and Pt 4f_7/2_ XPS spectral assignments. When comparing the estimated Pd 3d_5/2_ binding energies with reference data [65], we concluded that in SnO_2_/Pd nanocomposite, palladium was present in the +2 oxidation state corresponding to the PdO. The Pd3d XP spectrum of the nanocomposite SnO_2_/PtPd was shifted toward lower energies (Figure 8a), indicating a partial reduction of Pd. However, it was not possible to decompose the spectrum into two components that corresponded to different palladium oxidation states (0, +2). The Pt4f XP spectra of both SnO_2_/Pt and SnO_2_/PtPd nanocomposites corresponded to the Pt + 2 oxidation state in PtO [66]. When taking into account how the depth of the XPS analysis was determined by the mean free path of electrons with respect to inelastic collisions, and is 0.5–2.5 nm for metals and 4–10 nm for organic substances, it was impossible to exclude the presence of Pd^0^ and especially, Pt^0^ inside the particles (under the oxide layer).

The O1s XP spectra consisted of two components (Figure 8c) corresponding to the oxygen anions in the SnO_2_ lattice (530.7–530.9 eV) and to different forms of chemisorbed oxygen and hydroxyl groups on the SnO_2_ surface (531.8–532.0 eV). The impact of the higher energy component is 23–27% and did not depend significantly on the type of modifier.

**Figure 9:**
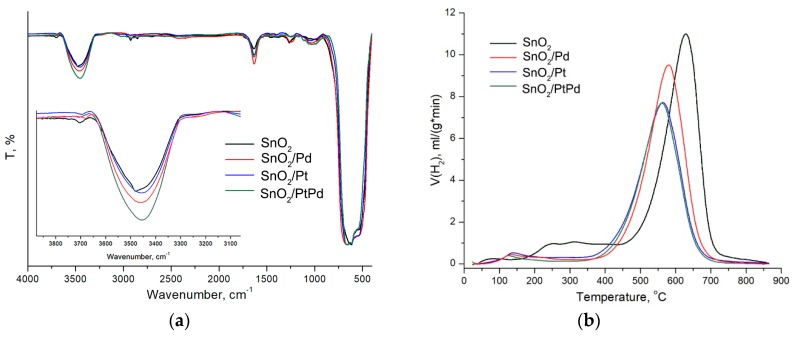
(**a**) IR spectra of blank SnO_2_ and nanocomposites normalized to the intensity Sn-O oscillations. Inset: enlarged spectra in 3100–3800 cm^−1^ region. (**b**) TPR-H_2_ profiles of blank SnO_2_ and nanocomposites.

The infra red (IR) spectra of blank SnO_2_ samples and nanocomposites are compared in Figure 9a. The intense absorption band at 400–800 cm^−1^ corresponded to the oscillations of Sn-O-Sn bridges (670 cm^−1^), Sn-OH terminal bonds (590 cm^−1^), and surface (540 cm^−1^) and bulk (480–460 cm^−1^) phonon vibrations of SnO_2_ [67]. The other absorption bands in the spectrum, apparently, were due to adsorbates. For a clear comparison of their concentration, the baselines were subtracted from the spectra, and the transmission in the whole range was normalized to the peak of the lattice vibrations at 670 cm^−1^. The broad absorption band at 3300–3650 cm^−1^, apparently referred to the stretching vibrations of O-H adsorbed water derivatives [68]. The modification of tin dioxide with palladium led to a significant increase in the concentration of surface hydroxyl groups, as evidenced by the increase in absorption in the range of 3300–3650 cm^−1^. On the contrary, the introduction of platinum did not have any effect on the concentration of hydroxyl groups on the SnO_2_ surface. For the SnO_2_/PtPd nanocomposite, a nonadditive increase in the concentration of OH groups was observed in comparison with SnO_2_/Pt and SnO_2_/Pd nanocomposites.

The results of the TPR-H_2_ experiments are shown in Figure 9b and in Table 3. The high-temperature (350–850 °C) peak corresponded to the hydrogen consumption because of SnO_2_ reduction to metallic tin:

(2) $${\mathrm{SnO}}_{2}+2H_{2}=\mathrm{Sn}+2H_{2}O$$

For nanocomposites SnO_2_/Pd, SnO_2_/Pt, and SnO_2_/PtPd, a shift was observed from the high-temperature maximum of hydrogen consumption to the lower temperature region. This may be due to the catalytic activity of noble metal clusters in the matrix of nanocrystalline tin dioxide. The most probable mechanism was the hydrogen and oxygen joint spillover [69] of the SnO_2_ crystal lattice through the clusters of modifiers. The reduction of SnO_2_ at a lower temperature became possible due to the dissociation of hydrogen molecules on noble metal clusters. Examples illustrating such a mechanism of interaction with the gas phase for different “noble metal/metal oxide” systems were provided in the review [69]. By the oxygen isotopic exchange method [70], it was established that the modification of nanocrystalline tin dioxide with palladium and ruthenium resulted in the realization of a multistage heteroexchange mechanism, involving the dissociation of the O_2_ molecule on the surface of the clusters of platinum-group metals, spillover of atomic oxygen from the clusters of modifiers to the SnO_2_ surface, and its rapid exchange with the oxygen of the crystal lattice of tin dioxide. According to the analysis of the oxygen isotopic exchange data presented in the review [71], platinum was more active in this process than palladium, explaining the more significant decrease in the temperature of the complete reduction of the SnO_2_/Pt and SnO_2_/PtPd nanocomposites compared to SnO_2_/Pd.

The processes responsible for the H_2_ consumption in low temperature region 100–300 °C can be expressed as follows

(3) $$O_{2\left(ads\right)}^{-}+2H_{2}=2H_{2}O+e^{-}$$

(4) $$O_{\left(ads\right)}^{-}+H_{2}=H_{2}O+e^{-}$$

(5) $${\mathrm{OH}}_{\left(ads\right)}+\frac{1}{2}H_{2}=H_{2}O$$

The amount of oxygen adsorbed on the surface of SnO_2_, estimated from TPR data under the assumption of interaction (3) is ~10^−4^ mol/m^2^. Assuming an orientation along the normal to the surface and a radius as in the $O^{-}$ (1.76 Å) ion, the coverage of SnO_2_ surface with hypothetical $O_{2\left(ads\right)}^{-}$ ions can be estimated at 0.5–1 monolayer. This is 2–3 orders of magnitude greater than the Weisz limitation for coverage of a semiconductor with charged adsorbates (10^−2^–10^−3^ monolayer [72]), indicating that the real composition of oxidative adsorbates on the surface of materials was much more diverse than this assumption.

Modification of the SnO_2_ surface with mono- and bimetallic clusters led to a decrease in the amount of hydrogen consumed in the low-temperature range. This indirectly indicated an increase in the fraction of the monatomic form of chemisorbed oxygen. Indeed, when comparing the reactions (3) and (4) surfaces with the same negative charge, predetermined by the Weitz limitation, it can be concluded that in the latter case, a smaller hydrogen consumption should be observed. In the case of nanocomposites containing palladium, the decrease in hydrogen absorption may be due to an increase in the concentration of hydroxyl groups detected by the IR spectroscopy and consequently, an increase in the contribution of the process (5).

The obtained information on the structure and surface composition of SnO_2_ and nanocomposites SnO_2_/Pd, SnO_2_/Pt, and SnO_2_/PtPd allowed us to explain the differences in their sensor properties to CO in dry (RH = 0%) and humid (RH = 20%) air.

*SnO_2_/Pd nanocomposite*

For the SnO_2_/Pd nanocomposite, the observed sensor response to CO in dry air was not unexpected and can be described within the framework of the concepts of electronic and chemical sensitization [2,5,11]. 

(i) Direct oxidation of CO gas molecules on the surface of PdO_x_ clusters resulted in a partial reduction of the modifier, while the fraction of Pd^0^ increases:

(6) $${\mathrm{PdO}}_{x}+{\mathrm{xCO}}_{\left(gas\right)}\to \mathrm{Pd}+{\mathrm{xCO}}_{2\left(gas\right)}.$$

This effect corresponded to the mechanism of electronic sensitization. The electron work function *φ* for reduced palladium surface was smaller than in the case of PdO_x_ and is *φ* = 4.8 eV [73], which was close to the work function for SnO_2_ (*φ* = 4.9 eV, [74]). Thus, as a result of the reduction of PdO_x_ clusters, a barrier was removed at the Pd/SnO_2_ interface, which led to a decrease in material resistance and the sensor response appearance.

(ii) Strong chemisorption of CO molecules on Pd^0^ was accompanied by a weakening of the intramolecular bond in CO and facilitated its break and further transformations of chemisorbed molecules:

(7) $${\mathrm{Pd}}^{0}+{\mathrm{CO}}_{\left(gas\right)}\to {\mathrm{Pd}}^{\delta +}-{\mathrm{CO}}^{\delta -}.$$

(iii) Modification with PdO_x_ clusters led to an increase in the SnO_2_ concentration of paramagnetic centers ·OH and rooted hydroxyl groups OH···OH that participated in the low temperature oxidation of chemisorbed CO molecules:

(8) $${\mathrm{CO}}_{\left(gas\right)}+{\mathrm{OH}}_{surf}\to {\mathrm{CO}}_{2\left(gas\right)}+H^{+}+e^{-}.$$

The increase in humidity led to a decrease in the sensitivity of SnO_2_/Pd in the low temperature region due to competitive adsorption of water molecules and blocking of active sites on the surface of SnO_2_ and PdO_x_ clusters [75].

*SnO_2_/Pt nanocomposite*

The low response of nanocomposites SnO_2_/Pt was caused by direct CO oxidation on PtO_x_ clusters. Based on the results of complementary investigations by in situ and *operando* X-ray absorption spectroscopy and *operando* FT-IR spectroscopy, D. Degler et al. found that CO oxidation mainly occurs at the PtO_x_ surface (Pt–*O*–Pt sites) [76]. This led to a decrease in the quantity of CO molecules that can react with the oxygen chemisorbed on SnO_2_ surface. The authors assumed that if platinum is introduced by impregnation after the calcination of the SnO_2_ matrix, it forms a separate oxide phase, which creates additional reaction sites not electronically coupled to the SnO_2_. In our investigation, this assumption could not be used since the introduction of PtO_x_ clusters led to the important (~10^3^ times) increase in SnO_2_ resistance in dry air (Figure 1a). The work function of metallic Pt was sufficiently high (*φ* = 5.65 eV [77]). Covering platinum with a full monolayer of oxygen led to the further increase in the work function by 1.19 eV (*φ* ~ 6.8 eV) [78]. When a contact was formed between PtO_x_ nanoparticles and SnO_2_ (*φ* = 4.9 eV), the Fermi level of the semiconductor oxide was pinned to the Pt^2+^/Pt^0^ potential. This led to the formation of an electron depleted space charge region and to an increase in the SnO_2_ resistivity. As in the case of PdO_x_, it was possible that PtO_x_ clusters reduced upon interaction with CO [79]. However, the work function of metallic Pt significantly exceeded the corresponding value for SnO_2_. As a result, the band-bending at the Pt/SnO_2_ interface persisted in the presence of CO, which led to a low sensor response of SnO_2_/Pt nanocomposite when CO was detected in dry air.

In humid air, the Pt–*O*–Pt sites were deactivated [76] because of strong water adsorption on PtO_x_ clusters [75]. This should have led to a decrease in the amount of CO oxidized directly on the clusters of the modifier, and consequently, to an increase in the sensor response due to an increase in the amount of CO oxidized by oxygen chemisorbed on the SnO_2_ surface. However, in humid air, the surface of tin dioxide also became inactive due to the partial replacement of chemisorbed oxygen by hydroxyl groups according to the following reaction [80]

(9) $$H_{2}O_{\left(gas\right)}+2\cdot {\mathrm{Sn}}_{\mathrm{Sn}}+O_{\left(ads\right)}^{\alpha -}\to 2\cdot \left(\mathrm{Sn}-\mathrm{OH}\right)+\alpha \cdot e^{-}$$

The combination of these factors suggests that CO oxidation can take place at the Pt–*O*–Sn sites at the three-phase boundary between PtO_x_ and SnO_2_ [76], providing a very slight increase in sensor response as compared with detection in dry air.

*SnO_2_/PtPd nanocomposite*

A feature of the SnO_2_/PtPd nanocomposite was a two-level distribution of the modifiers over the surface of the semiconductor matrix: platinum only existed as a part of large (20–60 nm) bimetallic particles PtPdO_x_ with a Pt content generally exceeding 50 mol%, while palladium was distributed between these large bimetallic particles PtPdO_x_ and small (~2 nm) particles PdO_x_. As a result of this distribution of modifiers, the active sites on the surface of SnO_2_/PtPd were a combination of the centers characteristic for SnO_2_/Pt and SnO_2_/Pd. Thus, the temperature of SnO_2_ reduction in the SnO_2_/PtPd nanocomposite coincided with that of SnO_2_/Pt (Figure 9b), which was due to the presence of particles enriched in platinum. At the same time, an increase in the concentration of hydroxyl groups on the surface of SnO_2_/PtPd (Figure 9a) was determined by the presence of small PdO_x_ particles.

However, when comparing the sensor response, such an additive picture was not observed. To explain the inversion of sensor response from *n*- to *p*-type observed in dry conditions, it was necessary to analyze the combined effect of the modifiers on the electrophysical properties of SnO_2_. Since metals Pt, Pd, and, to a greater extent, their oxides, are characterized by a higher work function than SnO_2_, in the contact areas SnO_2_-PtPdO_x_ and SnO_2_-PdO_x_, an electron-depleted layer formed in SnO_2_. The depth of this layer was determined using the height of the energy barrier—i.e., the difference in the work function values of the contacting materials—and the lateral length along the surface was determined by the area of the contact between SnO_2_ and clusters of modifiers. In the SnO_2_/PtPd nanocomposite, bimetallic particles enriched in platinum form on the surface of the SnO_2_ agglomerates regions with a deep depleted layer, while small PdO_x_ particles formed extended space charge regions of the surface of SnO_2_ grains. As a result, it can be expected that the concentration of electrons in SnO_2_ near the surface layer became so low that they ceased to be the main charge carriers. Such a change in the response type was reported for various oxides: from *n*- to *p*-type for MoO_3_ [81], In_2_O_3_ [82], SnO_2_(Fe) [83], ZnO [84], WO_3_ [85], WO_3_ nanorods [86], TiO_2_ nanofibers [87], and from *p*- to *n*-type conductivity for α-Fe_2_O_3_ [88]. The inversion of the sensor response was explained by a change in the type of main charge carriers in semiconductor oxide due to either the surface reactions under certain conditions, or because of the effect of impurities.

In dry air, in the high temperature region, the *p*-type response of the SnO_2_/PtPd nanocomposite had a similar temperature dependence as the *n*-type response of the SnO_2_/Pd nanocomposite. It indicated that under these conditions, the PdO_x_ small nanoparticles determined the reactivity of SnO_2_/PtPd sample through reaction (6). The reduced amplitude of the *p*-type response of SnO_2_/PtPd nanocomposite compared with the *n*-type response of SnO_2_/Pd was because of the direct oxidation of part of the CO molecules on the surface of large bimetallic PtPdO_x_ particles. This process did not alter the concentration of charge carriers in the SnO_2_ semiconductor matrix.

In humid air, the adsorption of water vapor on SnO_2_, in addition to blocking the active centers, led to an increase in the concentration of electrons and in the conductivity of SnO_2_ (reaction (9)). Comparison of the data presented in Figure 1a,b, clearly demonstrates that an increase in the air relative humidity (25 °C) from RH = 0% to RH = 20% reduced the resistance of all samples by about 10 times at each measurement temperature. Thus, in a humid atmosphere, electrons remained the main charge carriers in the SnO_2_/PtPd nanocomposite and no inversion of sensor response from *n*- to *p*-type was observed.

## 3. Materials and Methods

The synthesis of nanocrystalline SnO_2_ was carried out using flame spray pyrolysis (FSP). First, 20 mL of tin (II) 2-ethylhexanoate was dissolved in 60 mL of toluene; the resulting solution was divided into four equal parts, each of them then slowly injected into the FSP reactor. After the completion of each injection, the apparatus was dismantled, tin dioxide powder was collected, and a clean filter was installed. The obtained portions of the powder were combined and annealed in air at 400 °C for 24 h.

For the modification by impregnation method, the solutions of Pt (II) acetylacetonate and Pd (II) acetylacetonate in ethanol were used as metal precursors. The calculated volume of the precursor solution was added to the weighed SnO_2_ powder to obtain 1 wt.% total metal content (1 wt.% M for monometallic nanocomposites and 0.5 wt.% Pd + 0.5 wt.% Pt for bimetallic one) and ethanol was allowed to evaporate. All impregnated samples were then annealed at 300 °C for 24 h for decomposition of acetylacetonates. These annealing conditions corresponded to the lowest temperature, which ensured complete decomposition of both Pd(acac)_2_ and Pt(acac)_2_ that was proven using thermal analysis. A reference sample of SnO_2_ was created using an annealing undoped matrix material at 300 °C for 24 h.

The elemental composition of mono- and bimetallic nanocomposites was determined by X-ray fluorescence (XRF) analysis using a M1 Mistral (Bruker) micro-X-ray spectrometer. The phase composition of the samples was determined by XRD using a DRON-4-07 diffractometer (CuK_α_, λ = 1.5406 Å). The crystallite size of SnO_2_ phase (*d_XRD_*) was calculated by the Sherrer formula using 110 and 101 reflections.

The specific surface area was measured on Chemisorb 2750 instrument (Micromeritics) using a low-temperature nitrogen adsorption using single point BET model.

The microstructure of the samples and distribution of modifiers in SnO_2_ matrix were investigated using transmission electron microscopy (TEM), high angle annular dark field scanning transmission electron microscopy (HAADF-STEM), and energy dispersive X-ray (EDX) mapping, all accomplished using a FEI Osiris microscope equipped with a Super-X detector operated at 200 kV.

The chemical state of the elements was studied by X-ray photoelectron spectroscopy (XPS). The measurements were effectuated on K-Alpha (Thermo Scientific) spectrometer equipped with a monochromatic Al K_α_ X-ray source (Е = 1486.7 eV). The positions of the peaks in the binding energy scale were adjusted with a C1s peak (285.0 eV) that corresponded to the carbon contamination of the surface with an accuracy of 0.1 eV. XP-spectra were fitted by Gaussian–Lorentzian convolution functions with simultaneous optimization of the background parameters.

Active surface sites with oxidizing properties were investigated using the thermo-programmed reduction with hydrogen (TPR-H_2_) method. The experiments were carried out on Chemisorb 2750 (Micromeritics) in a quartz reactor at a gas mixture flow of 10% H_2_ in argon at 50 mL/min and at a heating rate of 10 °C/min to 900 °C.

The molecules adsorbed on the surface of materials were studied using the Fourier Transform infrared spectroscopy (FTIR) method. The IR spectra of the samples were taken on a Spectrum One (Perkin Elmer) spectrometer in transmission mode within the wavenumber range 400–4000 cm^−1^ with 1 cm^−1^ steps. The powders (5 mg) were grinded with 100 mg of dried KBr (Aldrich, “for FTIR analysis”) and pressed into tablets.

To perform the sensor tests, the powders were deposited onto microelectronic transducers equipped with Pt contacts and heaters in form of thick films. The sensors were placed into a gas flow chamber under conditions of a controlled gas flow of 100 ± 0.1 mL/min and operated by a resistance-measuring device connected to a PC. The DC-resistance was registered in situ under changing conditions in a temperature range of 60–300 °C. CO-air mixture containing 6.7 ppm CO was used as a test gas. The required level of humidity (RH = 0% and RH = 20%) was provided by mixing two streams of dry air and humid air using the membrane humidifier Cellkraft P-2.

## 4. Conclusions

The nanocrystalline SnO_2_ was synthesized using flame spray pyrolysis and used as semiconductor matrix to obtain the mono- and bimetallic nanocomposites SnO_2_/Pd, SnO_2_/Pt and SnO_2_/PtPd. It was found that in monometallic nanocomposites SnO_2_/Pt and SnO_2_/Pd, platinum forms large (several tens of nanometers) particles, PtO_x_, while palladium was distributed as small (including less than 2 nm) PdO_x_ nanoparticles on the SnO_2_ surface. In bimetallic nanocomposite SnO_2_/PtPd, platinum was located in large bimetallic PtPdOx particles with a different Pt/Pd ratio, but palladium was also present in the form of small nanoparticles PdO_x_.

The surface characteristics of bimetallic SnO_2_/PtPd nanocomposite were not additive as compared with monometallic SnO_2_/Pt and SnO_2_/Pd samples. Thus, active surface sites with oxidizing properties were determined by the presence of Pt-containing particles, while PdO_x_ nanoparticles were responsible for the increase in the surface hydroxyl concentration.

The sensor properties of bimetallic SnO_2_/PtPd nanocomposite can be explained by the combined effect of modifiers on the electrophysical properties of SnO_2_. The inversion of the sensor response from *n*- to *p*-type observed for SnO_2_/PtPd nanocomposite in dry air was due to a change in the type of main charge carriers in the near-surface layer of SnO_2_. Furthermore, the chances was due to the formation of a deep and extended electron depleted layer in the area of SnO_2_ contacts with platinum-enriched bimetallic PtPdO_x_ particles and PdO_x_ nanoparticles. In humid air, the adsorption of water vapor on SnO_2_ led to an increase in the concentration of electrons. As a result, electrons remained the main charge carriers and no inversion of sensor response was observed.

## Figures and Tables

**Table 1:** Microstructure characteristics and composition of investigated samples.

Sample	S_surf_, m^2^/g	*d_XRD_* (SnO_2_), nm ^(*a*)^	*d_TEM_*, nm ^(*b*)^			[M], wt.% ^(*c*)^
			SnO_2_	Pd	Pt	
SnO_2_	22 ± 5	10 ± 1	10.7 ± 4.9	-	-	-
SnO_2_/Pd				<2; 8–20	-	1.5 ± 0.2 ^(*c*)^
SnO_2_/Pt				-	25–100	1.0 ± 0.2 ^(*c*)^
SnO_2_/PtPd				<2	17–64	1.3 ± 0.2 (Pd) 0.3 ± 0.1 (Pt)

^(*a*)^ crystallite size (X-ray diffraction, XRD); ^(*b*)^ particle size (transmission electron microscopy, TEM); ^(*c*)^ obtained using X-ray fluorescence (XRF) analysis.

**Table 2:** Pd 3d_5/2_ and Pt 4f_7/2_ XP spectral assignments.

Spectral Assignment	Binding Energy, eV				
	Ref. [65]	Ref. [66]	SnO_2_/Pd	SnO_2_/Pt	SnO_2_/PtPd
Pd 3d_5/2_	Pd (0) 335.4		336.9	-	336.0
	PdO 336.4				
Pt 4f_7/2_		Pt (0) 71.0	-	72.0	72.2
		Pt (II) 72.4			
		Pt (IV) 74.9			

**Table 3:** Results of the TPR-H_2_ experiments.

Sample	Hydrogen Consumption, Mol H_2_ per 1 Mol SnO_2_			T_max_, °C	$N\;(O_{2\left(ads\right)}^{-}),\;\mathrm{Mol}/m^{2}$
	Total	100–300 °C	370–850 °C		
SnO_2_	2.9 ± 0.3	0.7 ± 0.1	2.2 ± 0.5	630	1.0×10^−4^
SnO_2_/Pd	2.5 ± 0.3	0.2 ± 0.1	2.3 ± 0.5	580	3.0×10^−5^
SnO_2_/Pt	2.3 ± 0.3	0.4 ± 0.1	1.9 ± 0.5	565	6.0×10^−5^
SnO_2_/PtPd	2.1 ± 0.3	0.2 ± 0.1	1.9 ± 0.5	565	3.0×10^−5^
